# CLAVATA3 Signaling Buffers Arabidopsis Shoot Apical Meristem Activity in Response to Photoperiod

**DOI:** 10.3390/ijms25179357

**Published:** 2024-08-29

**Authors:** Jennifer C. Fletcher

**Affiliations:** 1 Plant Gene Expression Center, United States Department of Agriculture-Agricultural Research Service, Albany, CA 94710, USA; jfletcher@berkeley.edu; 2 Department of Plant and Microbial Biology, University of California, Berkeley, CA 94720, USA

**Keywords:** Arabidopsis, carpel, CLV, meristem, peptide, photoperiod, rosette, signaling, stem cells

## Abstract

Land plants grow throughout their life cycle via the continuous activity of stem cell reservoirs contained within their apical meristems. The shoot apical meristem (SAM) of Arabidopsis and other land plants responds to a variety of environmental cues, yet little is known about the response of meristems to seasonal changes in day length, or photoperiod. Here, the vegetative and reproductive growth of Arabidopsis wild-type and *clavata3 (clv3)* plants in different photoperiod conditions was analyzed. It was found that SAM size in wild-type Arabidopsis plants grown in long-day (LD) conditions gradually increased from embryonic to reproductive development. *clv3* plants produced significantly more leaves as well as larger inflorescence meristems and more floral buds than wild-type plants in LD and short-day (SD) conditions, demonstrating that CLV3 signaling limits vegetative and inflorescence meristem activity in both photoperiods. The *clv3* phenotypes were more severe in SDs, indicating a greater requirement for CLV3 restriction of SAM function when the days are short. In contrast, *clv3* floral meristem size and carpel number were unchanged between LD and SD conditions, which shows that the photoperiod does not affect the regulation of floral meristem activity through the CLV3 pathway. This study reveals that CLV3 signaling specifically restricts vegetative and inflorescence meristem activity in both LD and SD photoperiods but plays a more prominent role during short days.

## 1. Introduction

Stem cell reservoirs within shoot and floral meristems are the source of all cells that generate the above-ground architecture of higher plants. The stem cells reside at the apex of the meristems in a region termed the central zone, and their identity and activity are sustained by the cells in the underlying organizing center [1]. Stem cell descendants continuously form organ primordia on the flanks of the meristem: leaves during the vegetative phase of development and flowers during the reproductive phase that follows the transition to flowering. In the model plant, *Arabidopsis thaliana*, stem cell homeostasis is maintained through intercellular signaling via the CLAVATA (CLV)–WUSCHEL (WUS) pathway [2]. The WUS transcription factor is produced in the organizing center and moves into the overlying central zone cells, where it maintains their identity as stem cells and induces the expression of the *CLV3* gene encoding a small, secreted polypeptide. Mobile CLV3 polypeptides are perceived by a suite of plasma membrane-associated receptors, including CLV1 and CLV2, that restrict the size of the *WUS* expression domain, forming a negative feedback loop that limits stem cell accumulation throughout the life cycle [3,4]. In addition to these intrinsic signals, the shoot apical meristem (SAM) responds to various environmental signals during plant growth and development, including light, nutrient availability, ambient temperature, and abiotic stress [5].

Environmental signal responsiveness by the SAM is particularly important at the transition to flowering, which occurs in response to both endogenous and environmental cues. Day length and other light cues are perceived in the leaves, and under inductive long-day (LD) photoperiod conditions, light signaling promotes flowering in Arabidopsis through the induction of *FLOWERING LOCUS T (FT)* and *TWIN SISTER OF FT (TSF)* expression in leaf vascular cells [6]. FT protein, the florigen molecule, is transported from the leaves to the SAM [7,8,9], where it physically associates with FLOWERING LOCUS D (FD) and 14-3-3 proteins [10,11]. This protein complex then up-regulates the expression of hundreds of genes, including *SUPPRESSOR OF OVEREXPRESSION OF CONSTANS1 (SOC1)* and *APETALA1 (AP1)*, that confer floral identity on the primordia that subsequently arise on the flanks of the meristem [12]. Under non-inductive short-day (SD) conditions, the activity of the SHORT VEGETATIVE PHASE (SVP) MADS domain transcription factor [13] reduces *FT, TSF,* and *SOC1* transcription, delaying flowering [14,15,16]. These pathways help synchronize SAM activity with the changing seasons.

In many plant species, the SAM undergoes a change in geometry during the floral transition, and its mitotic activity increases, especially in the central zone [17,18]. During the Arabidopsis floral transition, the SAM assumes a domed shape, and both the number and size of the cells in the SAM increase [19]. Several factors that control flowering time, including FT/TSF1 and the GA signaling pathway, contribute at least transiently to this meristem size increase during doming [19]. A SAM size increase is likewise associated with floral induction in tomato, in which the *LATE TERMINATION (LTM)* gene coordinates SAM doming with the floral transition process by antagonizing the activity of the floral inhibitor gene *SELF PRUNING* [20]. However, the precocious doming observed in *ltm* plants is not accompanied by the altered regulation of *CLV3* or *WUS* expression [20], leaving open the question of what effect, if any, environmental signals that are received and integrated by the SAM during the transition to flowering have on the CLV-WUS signaling pathway.

The behavior of CLV pathway components under different photoperiod conditions remains poorly understood. Two previous studies comparing the flowering time and floral meristem activity of *clv* mutant plants in the Landsberg *erecta* (L*er*) accession grown in short-day (SD) versus continuous light conditions showed that their floral meristem defects were suppressed in SD conditions [21,22]. However, L*er* contains a mutation at the *FLC* locus that accelerates flowering, potentially minimizing the differences between genotypes, and *clv3* null alleles were not consistently analyzed. To determine the effect of the photoperiod on CLV pathway function in the shoot apical meristem, the vegetative and reproductive phenotypes of two *clv3* null alleles in the Columbia-0 (Col-0) accession, *clv3-10* and *clv3-15*, grown to maturity in either long-day or short-day conditions, were analyzed. It was determined that *clv3* vegetative meristems (VMs) produced lateral organs at a faster rate than wild-type VMs in both LD and SD conditions and that *clv3* inflorescence meristems (IFMs) grew larger and produced more floral buds. Growth in SDs accelerated *clv3* leaf and floral bud production even more than growth in LDs. Further, SD growth conditions failed to suppress the flower phenotypes of *clv3* plants in the Col-0 accession. These results demonstrate that day length has a significant influence on CLV3-mediated meristem activity during vegetative and inflorescence development.

## 2. Results

**Figure 1:**
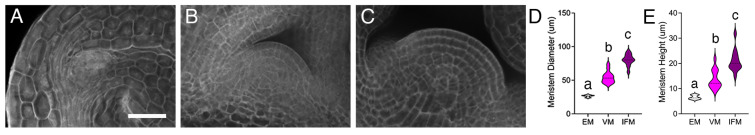
Shoot apical meristem size increases over time in wild-type plants grown in long-day conditions. (**A**) Col-0 embryonic meristem (EM), (**B**) Col-0 vegetative meristem at 7 days after germination (VM), and (**C**) Col-0 inflorescence meristem (IFM) at bolting. (**D**) Mean meristem diameter and (**E**) mean meristem height. Lower case letters indicate statistically significant differences (*p* < 0.001). n = 12–14. Scale bar, 25 µm.

To investigate the environmental effect of the photoperiod on *CLV3*-mediated shoot and floral meristem activity, the hypothesis that wild-type Arabidopsis SAM size increases during development was tested. Wild-type Col-0 plants grown under LD conditions at the embryonic, vegetative, and reproductive stages were collected and their SAMs analyzed using confocal laser scanning microscopy (Figure 1). The mean diameter of mature embryo SAMs was 26.4 µm, and the height was 6.5 µm (Figure 1A,D,E). By 7 days after germination (DAG), the average vegetative SAM had doubled in size to 54.6 μm wide and 13.7 µm tall (Figure 1B,D,E). Upon reaching the reproductive phase, the inflorescence meristems (IFMs) averaged 80.2 µm in diameter and 21.1 µm in height (Figure 1C–E), more than triple the dimensions of the mature embryos. These results indicate that the shoot apical meristem of wild-type Arabidopsis plants grows gradually but steadily under LD photoperiod conditions.

**Figure 2:**
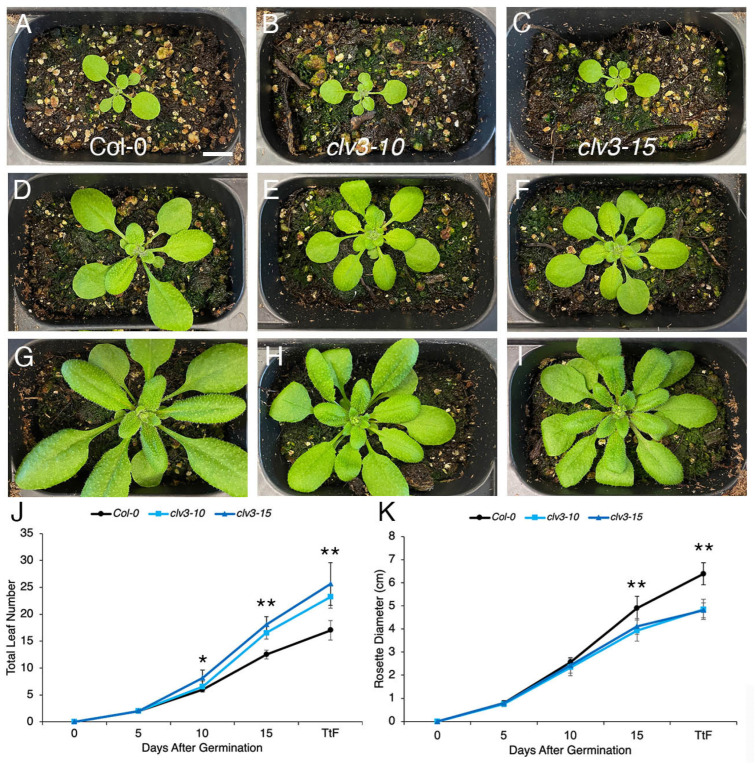
The leaf initiation rate and rosette diameter of wild-type and *clv3* plants grown in long-day conditions. A (**A**) Col plant, (**B**) *clv3-10* plant, and (**C**) *clv3-15* plant at 10 days after germination (DAG). A (**D**) Col plant, (**E**) *clv3-10* plant, and (**F**) *clv3-15* plant at 15 DAG. The (**G**) Col plant, (**H**) *clv3-10* plant, and (**I**) *clv3-15* plant at the transition to flowering (TtF). The (**J**) mean total leaf number and (**K**) mean rosette diameter +/− standard deviation (S.D.). Asterisks denote statistically significant differences at * *p* < 0.05 and ** *p* < 0.0001. n = 22. Scale bar, 1 cm.

Next, the effect of the photoperiod on the meristem activity of wild-type and *clv3* null mutant plants was examined. The plants were grown in either LD or SD conditions, and vegetative and reproductive meristem activity were assessed as measured by the number of leaves, floral buds, and carpels produced in response to the two divergent photoperiods. Up to five days after germination in LD conditions, the rosettes of Col-0, *clv3-10*, and *clv3-15* plants were indistinguishable from one another (Figure 2J,K). However, by 10 DAG, *clv3-10* and *clv3-15* vegetative meristems had generated one or two more leaves than wild-type meristems (Figure 2A–C,J). This trend continued as the plants aged, as by 15 DAG, *clv3-10* and *clv3-15* VMs had generated an average of 16–18 leaves compared to 12.5 for wild-type VMs (Figure 2D–F,J). At the transition to flowering, which occurred at 16-17 DAG under our growth conditions, Col-0 VMs produced an average of 17 leaves, a rate of one leaf per day, whereas *clv3-10* and *clv3-15* VMs produced an average of 24 leaves (Figure 2G–J). During the first 10 days of vegetative development, Col and *clv3* VMs generated new primordia at a rate of 0.6 and 0.7 leaves per day, respectively (Figure 2J). During the last 7 days of vegetative growth, Col and *clv3* VMs generated new primordia at a faster rate of 1.57 and 2.43 leaves per day, respectively, indicating that the plastochron of LD-grown plants shortens towards the latter part of vegetative development.

Unexpectedly, it was observed that the rosette diameter of 15 DAG *clv3-10* and *clv3-15* plants was significantly smaller than that of Col-0 plants (Figure 2D–F,K). This size difference became even more pronounced at the transition to flowering (Figure 2G–I,K). Therefore, beginning halfway through the vegetative phase, *clv3* meristems exhibit an accelerated rate of leaf initiation compared to wild-type meristems. This acceleration becomes particularly marked towards the end of vegetative development. In addition, the overall rosette size of *clv3* seedlings was smaller, producing more compact plants. These results indicate that CLV3 signaling not only limits the rate of leaf primordium initiation but also promotes rosette growth in LD conditions.

**Figure 3:**
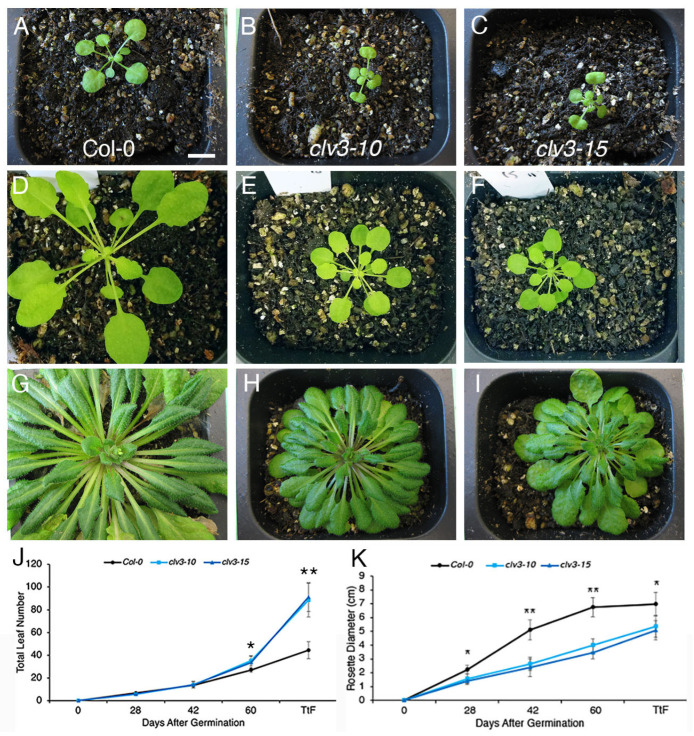
The leaf initiation rate and rosette diameter of wild-type and *clv3* plants grown in short-day conditions. A (**A**) Col plant, (**B**) *clv3-10* plant, and (**C**) *clv3-15* plant at 28 days after germination (DAG). A (**D**) Col plant, (**E**) *clv3-10* plant, and (**F**) *clv3-15* plant at 42 DAG. A (**G**) Col plant, (**H**) *clv3-10* plant, and (**I**) *clv3-15* plant at the transition to flowering (TtF). The (**J**) mean total leaf number and (**K**) mean rosette diameter +/− standard deviation (S.D.). Asterisks denote statistically significant differences at * *p* < 0.05 and ** *p* < 0.0001. n = 23–28. Scale bar, 1 cm.

These trends were more pronounced during the vegetative development of wild-type and *clv3* plants in SD conditions. For the first 42 DAG Col-0, *clv3-10* and *clv3-15* vegetative meristems generated similar numbers of rosette leaves (Figure 3A–C,J). However, after 42 DAG, *clv3-10* and *clv3-15* VMs began to generate significantly more leaves than wild-type VMs (Figure 3D–F,J). The rate of leaf initiation by *clv3* VMs then accelerated drastically from 60 DAG until the transition to flowering, which under our growth conditions occurred at a mean of 78 DAG for Col-0 and 90 DAG for *clv3* plants. At this time, Col-0 VMs had produced an average of 44.5 rosette leaves, whereas *clv3-10* and *clv3-15* VMs had produced twice that number, an average of 88.5 and 91.3 rosette leaves, respectively (Figure 3G–J). During the first 60 days of vegetative development in SDs, Col and *clv3* VMs generated new primordia at a rate of 0.45 and 0.57 leaves per day, respectively. During the remaining days of vegetative growth in SDs, Col VMs generated new primordia at a faster rate of 1.0 leaves per day, and *clv3* VMs generated new primordia at an even more accelerated rate of 1.86 leaves per day (Figure 3J). The rate of leaf formation therefore increases towards the latter stages of wild-type vegetative development in SD as well as LD conditions and accelerates even faster when CLV3 signaling is impaired.

Additionally, the reduction in rosette diameter observed in LD-grown *clv3* plants was more extreme under SD conditions. By 28 DAG, both *clv3-10* and *clv3-15* rosettes were already slightly yet significantly smaller than Col-0 rosettes (Figure 3A–C,K), and by 42 DAG, the *clv3* rosettes were half the diameter of the Col-0 rosettes (Figure 3D–F,K). The vegetative meristems of wild-type plants grown under SD conditions therefore initiate rosette leaves at a relatively constant rate of 0.56 leaves per day, whereas *clv3* meristems greatly accelerate leaf initiation toward the end of the vegetative phase. Moreover, the rosettes of *clv3* plants grown in SDs fail to grow to the extent of wild-type rosettes beginning a third of the way through vegetative development. In sum, CLV3 signaling limits the rate of leaf initiation in the vegetative meristem and promotes overall rosette growth under both LD and SD conditions, but the effects are more pronounced when the days are short.

**Figure 4:**
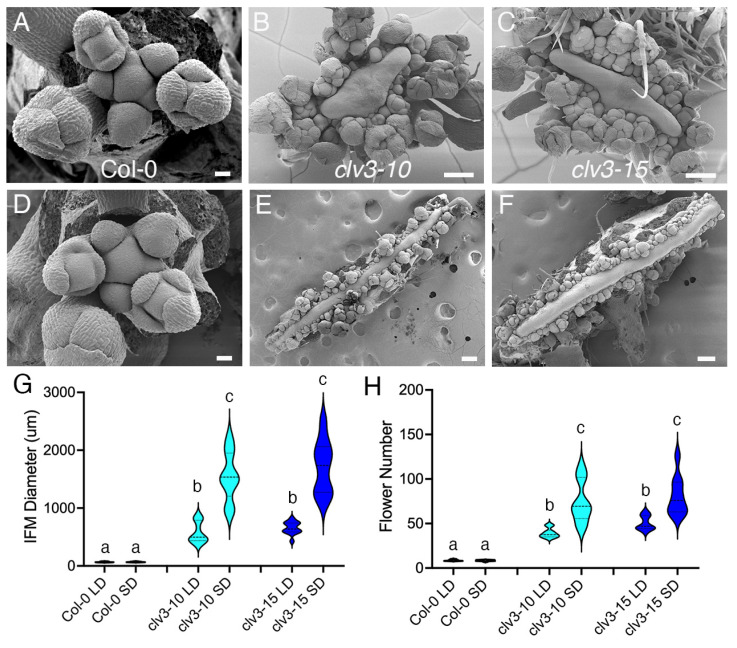
The inflorescence meristem activity of wild-type and *clv3* plants grown in long-day or short-day conditions. (**A**) Col, (**B**) *clv3-10*, and (**C**) *clv3-15* inflorescences in LD conditions and (**D**) Col, (**E**) *clv3-10*, (**F**) *clv3-15* inflorescences in SD conditions. The (**G**) mean inflorescence meristem (IFM) diameter in LD and SD conditions. The (**H**) mean stage 1-5 floral bud number per IFM in LD and SD conditions. Lower case letters indicate statistically significant differences (*p* < 0.001). n = 9–14. Scale bar, 20 µm (**A**,**D**), 100 µm (**B**,**C**,**E**,**F**).

Following the transition to flowering, the shoot apical meristem becomes an inflorescence that initiates floral meristems (FMs) rather than leaves on its flanks. *clv3* inflorescence meristems (IFMs) are far larger than wild-type meristems at the reproductive stage of development [23,24], yet whether *clv3* IFM activity is affected by day length has not been studied. To analyze this, Col-0 and *clv3* plants were grown in LDs or SDs, and IFMs were collected just after the transition to flowering to quantify IFM size and the number of stage 1-5 FMs produced in the two conditions. Under LD conditions, Col-0 IFMs averaged 65.88 µm in diameter and generated an average of 8.55 stage 1-5 floral buds (Figure 4A,G,H). *clv3* IFMs were significantly enlarged, averaging over 500 µm in diameter and generating over 40 stage 1-5 floral buds (Figure 4B,C,G,H). The mean IFM diameter and mean floral bud number of Col-0 plants grown under SDs were indistinguishable from those grown under LDs (Figure 4A,D,G,H). In contrast, the *clv3-10* and *clv3-15* mean IFM diameter and mean floral bud number was far greater under SD (Figure 4E–H) than LD conditions. Therefore, CLV3 activity buffers IFM size as well as flower production under both photoperiods, playing a more prominent role when the days are short.

**Figure 5:**
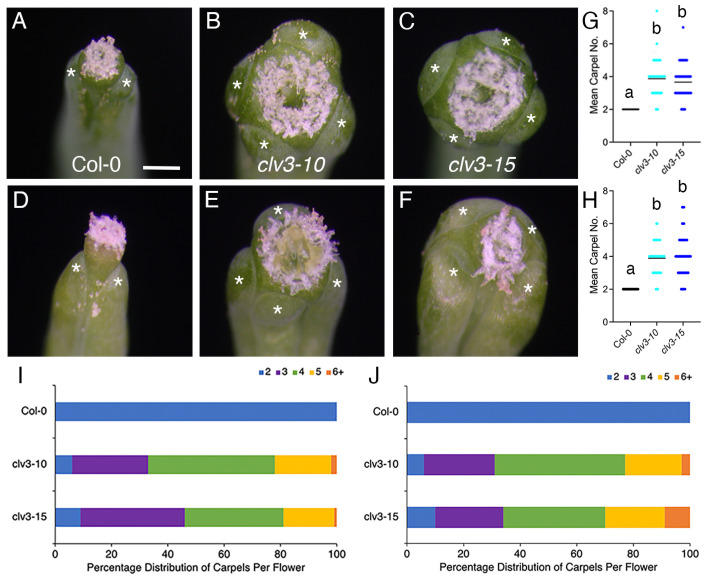
The carpel number of wild-type and *clv3* plants grown in long-day or short-day conditions. (**A**) Col, (**B**) *clv3-10*, and (**C**) *clv3-15* carpels in LD conditions and (**D**) Col, (**E**) *clv3-10*, and (**F**) *clv3-15* carpels in SD conditions. (**G**,**H**) The mean carpel number in (**G**) long-day and (**H**) SD conditions. (**I**,**J**) The percentage distribution of carpels per flower in (**I**) LD and (**J**) SD conditions. White asterisks denote carpels. Black bars indicate the mean. Lower case letters indicate statistically significant differences (*p* < 0.001). n = 100 gynoecia per genotype. Scale bar, 1 mm.

The final meristem type produced by many flowering plants is the floral meristem. Like the IFMs, the FMs of *clv3* plants are larger than wild-type FMs and generate more floral organs of each type, especially carpels [23,24]. To investigate the effect of the photoperiod on FM activity in *clv3* plants, the stage 4 FM size and carpel number was quantified in Col-0 and *clv3* plants grown under LD or SD conditions. Under LD conditions, wild-type Col-0 FMs averaged 55.93 µm in diameter and were only slightly larger under SD conditions (57.46 µm, Figure S1). Similarly, the *clv3-10* and *clv3-15* FM diameter, although enlarged compared to Col-0, was relatively unchanged in SD compared to LD conditions (Figure S1). The most sensitive readout of FM activity in *clv* mutants is the carpel number [25]. In LD conditions, wild-type Col-0 flowers produced 2 carpels whereas *clv3* flowers produced an average of 4 and a range of 2–8 carpels (Figure 5A–C,G,I). Almost identical phenotypes were observed under SD conditions (Figure 5D–F,H,J). The proportions of *clv3* flowers that generated a specific number of carpels was also very similar between LD (Figure 5I) and SD (Figure 5J) conditions. Thus, in contrast to vegetative and inflorescence meristem activity, the floral meristem activity in *clv3* plants is relatively unaffected by the photoperiod.

## 3. Discussion

Although the shoot apical meristem activity of land plants is influenced by a variety of environmental cues, including light, little is known about how the SAM responds to day-length signals by altering its behavior at the physiological and molecular levels. Here, it was observed that the shoot apical meristem size in wild-type Arabidopsis Col-0 plants grown in LD photoperiod conditions continually increased from embryonic to vegetative to reproductive development (Figure 1), consistent with the gradual increase in SAM area observed in wild-type Col-0 plants grown under SD conditions [19]. A gradual increase in SAM size during vegetative development in LD conditions was likewise observed in tomatoes [20] and in rice [26], suggesting that this growth pattern may be a common feature of land plant meristems.

The leaf initiation rate of vegetative meristems from wild-type plants grown in LD versus SD photoperiods was quantified. Col-0 plants grown in 21 °C, LD conditions produced approximately one leaf per day (Figure 2J), a nearly linear rate of leaf production also reported elsewhere [27]. In contrast, wild-type plants grown in SDs produced an average of 0.56 leaves per day (Figure 3J). These data indicate that fewer hours of daylight, such as during the fall and winter seasons, reduce the rate at which Arabidopsis plants initiate leaves.

Next the largely unexplored question of whether the activity of the CLV signaling pathway that regulates SAM maintenance is affected by changes in the photoperiod was investigated. It was found that *clv3* VMs initiated leaves at a significantly faster rate than wild-type VMs under both LD and SD photoperiods (Figure 2 and Figure 3). The leaf initiation rate of both wild-type and *clv3* VMs accelerated over time, but the rate of increase in *clv3* VMs was greater than that of Col-0 VMs. This resulted in the production of 1.4 times as many leaves in *clv3* plants than wild-type plants in LDs and twice as many leaves in *clv3* plants than wild-type plants in SDs. Thus, *clv3* seedlings produced significantly more leaves than wild-type seedlings at bolting, particularly under SD conditions (Figure 2J and Figure 3J). Therefore, short-day photoperiods increase the requirement for CLV3 signaling to restrict leaf production. Consistent with these results, additional rosette leaves were also produced under LD conditions by plants mutant for *Atrlp10-1,* a T-DNA insertion null allele of *clv2,* compared to Col-0 plants as well as by *clv2-3* null mutant plants compared to L*er* plants [28]. These data indicate that CLV3 signaling to limit rosette leaf initiation, at least in LDs, is likely mediated by the CLV2 receptor-like protein.

The leaf initiation rate is the inverse of the plastochron, which defines the time between the initiation of two successive leaf primordia and corresponds to the temporal pattern of leaf formation [29]. The data demonstrate that the plastochron of wild-type plants grown in LDs (24 h; Figure 2J) is shorter than that of plants grown in SDs (42 h; Figure 3J). Likewise, the plastochron of *clv3* plants grown in LDs (17 h; Figure 2J) is shorter than that of plants grown in SDs (21 h; Figure 3J). In addition, the plastochron of both wild-type and *clv3* plants shortens towards the end of the vegetative stage under both LD and SD conditions, when both genotypes make leaves at a faster rate (Figure 2J and Figure 3J). It should be noted, however, that the change in plastochron that occurs between SD and LD conditions is less pronounced for *clv3* VMs than for wild-type VMs: the plastochron of the average *clv3* VM lengthens from 17 h in LDs to 24 h in SDs, a 29.1% increase, whereas the plastochron of the average Col-0 VM lengthens from 24 h in LDs to 42 h in SDs, a 42.9% increase. Therefore, the requirement for CLV3 signaling to limit vegetative meristem activity is greater when the days are short.

A previous study reported that wild-type Col-0 and Ws-4 plants grown continuously in LDs formed smaller IFMs and slightly fewer floral primordia than those grown for one month in SD and then switched to LD conditions [30]. In addition, Col-0 plants had both larger IFMs and a longer plastochron than Ws-4 plants grown under the same light conditions. From these observations, it was inferred that meristem size might be negatively correlated to the plastochron [30]. However, data presented here that *clv3* plants with larger vegetative meristems [23,31] have a shorter plastochron than Col-0 plants in both LD and SD photoperiods suggest that this is not necessarily the case, at least not during the vegetative phase.

Arabidopsis is a facultative LD species that flowers later in SD than in LD conditions. Thus, a simple explanation for why both wild-type and *clv3* meristems produce more leaves in SD than in LD conditions is that the plants spend more time in the vegetative phase when the days are short, increasing the total number of leaves produced by the meristem. However, this implies that the leaf initiation rate is similar between LD and SD grown plants. This was not observed to be the case; instead, Col-0 plants grown in LD conditions displayed an overall leaf initiation rate of one leaf per day, whereas plants grown in SDs displayed a slower leaf initiation rate of 0.56 leaves per day (Figure 2J and Figure 3J). Further, the duration of the vegetative phase alone is likely insufficient to account for the accelerated rate of leaf initiation by *clv3* meristems because the number of days to flowering and the number of leaves are uncoupled from one another in many flowering time mutants [32], including *altered meristem program1 (amp1)* mutants that produce abnormally high levels of the phytohormone cytokinin [33]. *amp1* plants have enlarged meristems and a shorter plastochron [33,34], similar to *clv3* plants.

This study also revealed that *clv3-10* and *clv3-15* null mutant plants display significantly smaller rosettes than wild-type Col-0 plants. A reduced rosette diameter of *clv3* plants grown in LDs was observed just after the midway point of vegetative development (Figure 2) and was more severe when the plants were grown in SDs (Figure 3). A reduced rosette size phenotype was also noted in *clv3-9* null mutant plants grown under LD conditions [35]. This finding was unexpected because *CLV3* expression is specific to the stem cells in the shoot and floral meristems [31], and there is currently no evidence that CLV3 peptides, although mobile over short distances [36,37,38], are present in leaf primordia or expanding leaf tissues. Therefore, it can be hypothesized that the *clv3* rosette phenotype is an indirect consequence of the meristem phenotype, in which the accumulation of vegetative meristem cells [39] and the number of leaf primordia initiated (Figure 2J and Figure 3J) exceeds that of wild-type meristems.

Both light and energy metabolites are essential for organ formation by the Arabidopsis SAM [40]. Thus, a possible explanation for the reduced rosette size phenotype is that increased *clv3* SAM activity during the vegetative phase results in more metabolic resources than normal being allocated to meristem cell proliferation and primordium initiation and correspondingly fewer to leaf expansion. Metabolite reallocation between plant tissues has been reported during leaf senescence [41], in response to drought and salt stress [42,43], and in the transition from plant growth to defense [44,45]. Increased meristematic activity in *clv3* VMs may impose an enhanced demand for resources such as nitrogen [46] and sulfur [47] that might be redirected from the process of leaf outgrowth occurring simultaneously. Alternatively, or additionally, enhanced *clv3* VM activity might lead to the induction of abiotic stress response pathways that are known to affect Arabidopsis rosette size [48].

These metabolic or stress response pathways could potentially alter the activity of growth-regulating hormones [49,50,51], of transcription factors such as the GROWTH-REGULATING FACTORs (GRFs) [52,53], and/or of the highly conserved kinase TARGET OF RAPAMYCIN (TOR). The TOR complex serves as a central regulator of growth that integrates internal metabolic processes with external environmental cues, including biotic and abiotic stress [54,55]. When local environmental conditions are favorable, TOR is active and promotes growth by facilitating the biosynthesis and transport of amino acids, proteins, sugars, and other metabolites as well as inducing the expression of genes involved in growth hormone signaling [56]. In particular, increasing TOR activity increases leaf cell size and overall leaf size [57]. Conversely, when nutrients are limited or external stresses are present, the TOR kinase becomes inactivated, and growth is suppressed [54]. In the vegetative shoot apex, light and glucose are required to stimulate mitotic activity and leaf formation [40,58]. Together, these two cues activate TOR [40,58], which phosphorylates the E2Fa and E2Fb transcription factors to promote cell proliferation and leaf initiation [58]. TOR is also required during germination for the activation of *WUS* expression in the SAM by light and metabolic signals [40]. Further, TOR-dependent phosphorylation of the Polycomb Repressive Complex2 (PRC2) factor FERTILIZATION-INDEPENDENT ENDOSPERM (FIE) modulates the expression of *WUS* and other SAM regulator genes via the deposition of repressive histone methylation marks [59]. These collective activities make TOR a plausible candidate factor to coordinate CLV3-dependent meristem maintenance with rosette size control.

As it does with vegetative meristem activity, the CLV3 signaling pathway buffers inflorescence meristem activity from the effects of changes in day length. Both IFM size and floral bud production are relatively unaffected by the photoperiod in wild-type Col-0 plants yet are dramatically increased in *clv3* plants under LD and SD conditions (Figure 4). *clv3* plants grown in SDs have significantly more severe IFM phenotypes than those grown in LDs (Figure 4), indicating that the buffering role of CLV3 signaling during both inflorescence and vegetative development is more consequential when the days shorten. In contrast, CLV3 signaling has relatively little effect on FM size in different photoperiods (Figure S1), perhaps because FM activity is transient and of much shorter duration than VM and IFM activity [60].

Although little is known about the molecular mechanisms that link shoot meristem maintenance and photoperiod responses, there are several factors that may play a role. The FAR-RED ELONGATED HYPOCOTYL3 (FHY3) transcription factor is a key positive regulator of the Arabidopsis phytochrome A (phyA) signaling pathway [61], which senses short photoperiods [62]. FHY3 also promotes inflorescence meristem activity by directly repressing *CLV3* expression and is critical for the light-regulated expression of *CLV3* in the SAM [63]. This FHY3-phyA-CLV3 signaling module therefore may provide a mechanism for modulating meristem function in response to short days. Genes that induce changes in meristem size during the photoperiod-induced floral transition, such as *FT* [19] and *LTM* [20], may also contribute to coordinating meristem activity with day length.

Previous studies reported that SD growth conditions suppressed the increased carpel number phenotypes of *clv1, clv2,* and *clv3* null mutant plants, suggesting that the photoperiod regulates Arabidopsis floral meristem development [21,22,64]. *clv2* alleles displayed the most complete phenotypic suppression, whereas *clv3* alleles showed only partial suppression [21,64]. These studies predominantly used alleles in the fast-cycling L*er* background, although the *clv1-12* and *clv1-13* T-DNA insertion null alleles in Col-0 also displayed a partial suppression of carpel numbers in SDs [21]. In contrast, no difference in the carpel number between *clv3* Col-0 null mutant plants grown in LD or SD conditions was found in this analysis (Figure 5). This discrepancy may be partially accounted for by the difference in the genetic background. *erecta (er)* mutations, such as the one present in the L*er* background, increase shoot and floral meristem cell accumulation in a CLV-independent fashion [65,66,67,68]. As a result, the larger FMs of *clv3* L*er* plants might show a more pronounced response to photoperiod changes than the smaller FMs of *clv3* Col-0 plants. The involvement of the CLV2 receptor protein in developmental processes beyond the CLV3-mediated meristem maintenance pathway [22] may be an additional factor contributing to the enhanced sensitivity of the *clv2* alleles to changes in day length.

In sum, this study identifies a role for CLV3 signaling in restricting vegetative and inflorescence meristem activity during both LDs and SDs, with a more pronounced requirement when the days are short. This work therefore provides insight into how plant growth can be optimized in response to changing environmental conditions. Further investigation will be required to determine the underlying molecular mechanisms for this photoperiod-driven effect on meristem function.

## 4. Materials and Methods

All *Arabidopsis thaliana* lines were in the Columbia-0 (Col-0) background and have been previously described [24]. Seeds were sown in soil consisting of 50% medium vermiculite and 50% Sunshine Mix #1 (SunGro Horticulture, Agawam, MA, USA) and stratified for 5 days at 4 °C before being transferred to a growth chamber at 21 °C, 50% humidity under either long-day (16 h light: 8 h darkness) or short-day (8 h light:16 h darkness) conditions at 120 µmol m^−2^ s^−1^ light intensity. Following germination, the plants were fertilized daily with a dilute mixture of Miracle-Gro 20-20-20 fertilizer (Scotts Miracle-Gro Company, Marysville, OH, USA), 100 mg in 1 L water, until flowering.

For plants grown under long day conditions, rosette leaf number was measured every five days after germination and again at the transition to flowering. For plants grown under short day conditions, rosette leaf number was measured at 28, 42, and 60 DAG and again at the transition to flowering. The total leaf number and rosette diameter were measured at the transition to flowering when the first flower buds were visible. The total leaf number represented the sum of the rosette and cauline leaves. Rosette diameter measurements were made as the distance between the tips of the two longest rosette leaves at a 180-degree angle bisecting the shoot apex. The rate of leaf initiation was tested for linear growth using the formula *P(t)* = *P*_0_ + *td*, where *P* is the population leaf number, *P*_0_ is the initial population leaf number at germination, *t* is the time period, and *d* is the constant amount of change. Statistical analysis was performed using two-tailed, unpaired Student’s t-tests. Each experiment was performed twice, with similar results.

Embryo, vegetative, and inflorescence meristems were collected and fixed, stained with propidium iodide, cleared and mounted for confocal laser scanning microscopy, and measured as described [69]. For reproductive stage measurements, inflorescences were harvested after bolting 1 cm, just as the first floral bud opened, and fixed, dehydrated, dried, and mounted for scanning electron microscopy as described [69]. Each inflorescence was imaged, and inflorescence meristem diameter, stage 4 floral meristem diameter, and total number of stage 1-5 floral buds were quantified using the Fiji software (ImageJ2 v2.14.0, National Institutes of Health, Bethesda, MD, USA) straight line or multi-point tools. Carpel number measurements were performed as described [69]. Statistical analysis was performed using one-way ANOVA with post hoc Tukey’s test for multiple comparisons.

## Data Availability

Data are contained within the article and Supplementary Materials.

## Supplementary Materials

The following supporting information can be downloaded at https://www.mdpi.com/article/10.3390/ijms25179357/s1.
