# Supplementary material for: CLAVATA3 Signaling Buffers Arabidopsis Shoot Apical Meristem Activity in Response to Photoperiod
Source: Int J Mol Sci. 2024 Aug 29;25(17):9357. doi: 10.3390/ijms25179357 (PMC11394970; doi:10.3390/ijms25179357)
Supplement: Supplementary file 1 [file ijms-25-09357-s001.zip › ijms-3159068-supplementary.pdf]

Supplementary Materials

CLAVATA3 Signaling Buffers Arabidopsis Shoot Apical Meristem Activity in Response to Photoperiod

Jennifer C. Fletcher [1,2]

1 Plant Gene Expression Center, United States Department of Agriculture-Agricultural Research Service, Alba-ny, CA 94710, USA; jfletcher@berkeley.edu

2 Department of Plant and Microbial Biology, University of California, Berkeley, CA 94720, USA

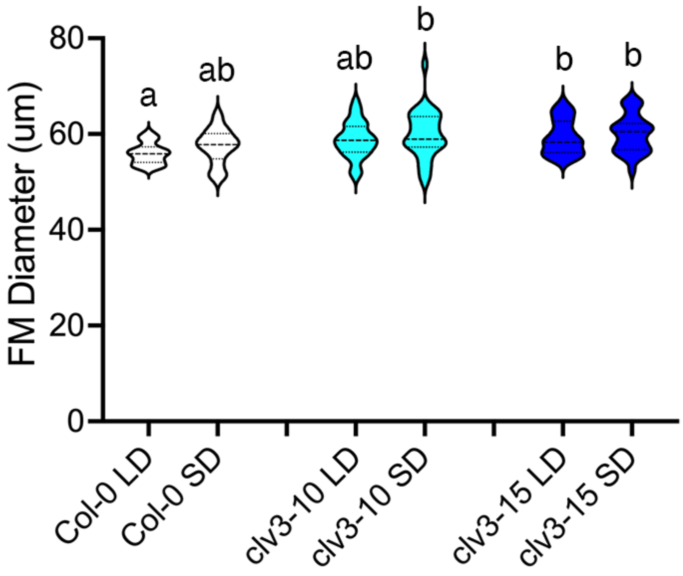

Figure S1. Mean stage 4 floral meristem (FM) diameter in LD and SD conditions. Lower case letters indicate statistically significant differences ($p < 0.001$). n = 13 to 36 floral meristems from 9 to14 plants per genotype.
